# Nitric Oxide Donors as Neuroprotective Agents after an Ischemic Stroke-Related Inflammatory Reaction

**DOI:** 10.1155/2013/297357

**Published:** 2013-04-04

**Authors:** Marisol Godínez-Rubí, Argelia E. Rojas-Mayorquín, Daniel Ortuño-Sahagún

**Affiliations:** ^1^Laboratorio de Desarrollo y Regeneración Neural, Instituto de Neurobiología, Departamento de Biología Celular y Molecular, CUCBA, Universidad de Guadalajara, camino Ing. R. Padilla Sánchez, 2100, Las Agujas, 44600 Zapopan, JAL, Mexico; ^2^Departamento de Ciencias Ambientales, Instituto de Neurociencias, CUCBA, Universidad de Guadalajara, 45100 Guadalajara, JAL, Mexico; ^3^Departamento de Investigación Básica, Instituto Nacional de Geriatría (INGER), Periférico Sur No. 2767, Col. San Jerónimo Lídice, Deleg. Magdalena Contreras, 10200 México, DF, Mexico

## Abstract

Cerebral ischemia initiates a cascade of detrimental events including glutamate-associated excitotoxicity, intracellular calcium accumulation, formation of Reactive oxygen species (ROS), membrane lipid degradation, and DNA damage, which lead to the disruption of cellular homeostasis and structural damage of ischemic brain tissue. Cerebral ischemia also triggers acute inflammation, which exacerbates primary brain damage. Therefore, reducing oxidative stress (OS) and downregulating the inflammatory response are options that merit consideration as potential therapeutic targets for ischemic stroke. Consequently, agents capable of modulating both elements will constitute promising therapeutic solutions because clinically effective neuroprotectants have not yet been discovered and no specific therapy for stroke is available to date. Because of their ability to modulate both oxidative stress and the inflammatory response, much attention has been focused on the role of nitric oxide donors (NOD) as neuroprotective agents in the pathophysiology of cerebral ischemia-reperfusion injury. Given their short therapeutic window, NOD appears to be appropriate for use during neurosurgical procedures involving transient arterial occlusions, or in very early treatment of acute ischemic stroke, and also possibly as complementary treatment for neurodegenerative diseases such as Parkinson or Alzheimer, where oxidative stress is an important promoter of damage. In the present paper, we focus on the role of NOD as possible neuroprotective therapeutic agents for ischemia/reperfusion treatment.

## 1. Introduction

When the brain blood flow is interrupted, it results in deprivation of oxygen and nutrients to the cells; this situation constitutes an ischemic stroke. Restoration of the flux, or reperfusion, can reduce the damage, but only when this is performed very early after the onset of ischemia, and its efficacy is restricted by secondary injuries, mainly by oxidative stress (OS) and an inflammatory reaction, which lead to cell death by apoptosis [1].

It is noteworthy that ischemic damage not only affects neurons. Thus, in recent years the concept of the neurovascular unit has been highlighted, emphasizing the need to protect not only the neurons, but also all cells in the brain [2–5]. In contrast to the known vulnerability of neurons and astrocytes, it is thought that endothelial cells tend to be more resistant to ischemic or oxidative injury [6]. Hence, to be successful, stroke therapies should be widely effective and must protect all neuronal, glial, and endothelial components in the brain [7]. 

After focal ischemia, primary neuronal death appears rapidly in the core area and is followed by secondary death in the ischemic penumbra, which evolves from the delayed activation of multiple cellular death pathways. At the core of the ischemic lesion, one of the first events is the rapid decline of adenosine triphosphate (ATP) reserves [8]. Consequently, all energy-dependent processes gradually cease their activity, leading to changes in transmembrane potential. The consequent depolarization (denominated anoxic depolarization) produces massive influx of Na^+^, Cl^−^, and Ca^2+^ inside the cell with K^+^ efflux [9].

Core anoxic ischemic depolarizations induce release of neurotransmitters such as glutamate. Once released, glutamate generates a phenomenon of peri-infarct depolarization, which increases energy consumption and promotes Ca^2+^ influx into the cells [10].

The increase in intracellular Ca^2+^ in neurons and glial cells initiates a set of nuclear and cytoplasmic events that produce deep brain tissue damage that includes the following: Ca^2+^ mitochondrial overload (which compromises the already affected ATP production and promotes the opening of the mitochondrial transition pore); the increase in OS, and the activation of a number of Ca^2+^-dependent enzymes. Such enzymes include proteases, kinases, phospholipases, and endonucleases, which destroy biomolecules [10]. Additionally, increased intracellular Ca^2+^ also promotes the production of NO from constitutive synthases that, together with acidosis and peri-infarct depolarization, contribute to the initiation of damage; later, inflammation and activation of apoptotic phenomena contribute to increased injury [2].

OS is a major mechanism implicated in stroke and in a variety of neurodegenerative diseases, mainly in Alzheimer and Parkinson (reviewed in [11, 12]). The most accepted theory regarding neurodegeneration in Parkinson disease refers to OS as the main cause of damage to neurons in the substantia nigra. In addition, in Alzheimer disease, the OS generated by the action of *β*-amyloid, which causes massive entry of Ca^2+^ and caspase activation, leads to neuronal death [13, 14]. 

During ischemia, reactive oxygen (ROS) and nitrogen species can be generated in the ischemic penumbra but can also be produced during reperfusion injury [15, 16]. Indeed, it is now established that albeit maintenance of partial or complete blood flow is essential for preserving cerebral tissue, it is during reperfusion when it paradoxically induces excessive generation of ROS, such as superoxide anion radical (O_2_^•−^), hydroxyl radical (OH^•^), hydrogen peroxide (H_2_O_2_), and nitric oxide (NO), which contribute to increased neuronal death by oxidizing proteins, damaging DNA, and inducing lipid peroxidation [17].

Reperfusion-induced ROS contribute to a decrease of the NO availability responsible for postischemic endothelial dysfunction [18, 19]. During the ischemic period, reduction in O_2_ availability reduces the activity of NO synthase, producing O^2−^ instead of NO; later, during reperfusion, the arrival of O_2_ increases NO synthase activity. These can exert a deleterious effect by promoting nitrosative stress and diminishing the availability of NO for preserving endothelial integrity. 

Over the past decade, remarkable advances have been made in understanding the basic molecular mechanisms underlying neuronal death. However, clinically effective neuroprotectants have not yet been discovered and no specific therapy for stroke is available at present. The body of experimental data supports the view that reducing OS should continue to be a potentially viable target for stroke therapy [20]. In addition, the inflammatory response requires consideration as a potential target of therapy for ischemic stroke [21]. Therefore, agents capable of modulating both elements will constitute promising therapeutic solutions [22–25].

## 2. Ischemic Lesion and Immune Response: Brain Inflammation

It has now been established that the Central nervous system (CNS) is able to raise an immune response to the majority of threatening stimuli, whereby resident cells generate inflammatory mediators including cytokines, prostaglandins, free radicals, complementary chemokines, and adhesion molecules that recruit immune cells and activate glia and microglia (reviewed in [21, 26–28]). The role of microglia and proinflammatory cytokines in the CNS has been characterized in models of brain insults, such as experimental stroke, the most common form of ischemic injury [26]. As mentioned previously, cerebral ischemia triggers acute inflammation, which exacerbates primary brain damage. Although inflammation should be adaptive, the release of proinflammatory cytokines has often been associated with harmful consequences to neurons and myelin [29].

The control of early CNS inflammation is a careful balancing act, as both too much and too little inflammation will lead to decreased or delayed recovery. Whether the inflammation is neurotoxic or protective may depend upon the context and the location of the inflammatory mediator in relation to an injury, and the timing of inflammatory response may determine the outcome (see Table 1 in [27]).

For example, tumor necrosis factor alpha (TNF-*α*) upregulated in the proximity of an evolving lesion contributes to secondary infarct growth, whereas cytokine induction remote from the ischemic lesion confers neuroprotection [30]. TNF-*α* could enhance apoptotic processes through its action on its tumor necrosis factor type 1 receptor (TNFR1) in models of acute (ischemia, excitotoxicity) and chronic (Alzheimer disease, multiple sclerosis) neurodegeneration [31]. TNF-*α* and interleukin 1beta (IL-1*β*) exert neurotoxicity in cerebral ischemia in the presence of elevated inducible nitric oxide synthase (iNOS), while in the absence of iNOS, both cytokines appear to contribute to neuroprotection and plasticity, highlighting the role of the context [32]. 

There is important recognition that protection of endothelial function and downregulation of vascular inflammation comprise part of neuroprotection phenomena and may possess added therapeutic benefit against stroke injury [33]. However, research on clinically effective neurovascular protective therapies for brain damage remains at an early phase [34]. Much attention has been focused on the role of NO in vessel protection from OS and inflammation [35]. Because OS coexists with inflammation and endothelial dysfunction, determining antioxidant status may be helpful in monitoring the progress of Nitric oxide donors (NOD) treatment. A variety of structurally different NOD, which release NO either as a free radical (NO^•^) or as an NO ion (NO^+^/NO^−^), have shown to reduce OS/inflammation and to increase cerebral blood flow [35–38]; thus, these can be considered attractive candidates for therapeutic agents in experimental models of stroke.

## 3. Nitric Oxide Donors (NOD) as Neuroprotective Agents in Ischemic Stroke

### 3.1. Nitric Oxide in Ischemic Stroke

Nitric oxide (NO) plays a dual role, that is, neuroprotection and neurotoxicity, in the pathophysiology of cerebral ischemia-reperfusion injury [39]. NO is synthesized by NOS, of which there are three known isoforms: nNOS; eNOS, and inducible or immunological NOS (iNOS). The first two are constitutively expressed and their activity is dependent on changes at the intracellular Ca^2+^ level, while iNOS acts in a Ca^2+^-independent manner [40]. Baseline concentration of NO in the brain is mainly due to nNOS activity, and secondarily to eNOS. iNOS is not expressed under physiological conditions [41, 42].

In the brain, eNOS is mainly produced by the vascular endothelium and the choroid plexus [43]. Although eNOS-NO production is a minor part of total brain NOS activity, this enzyme is critical for the regulation of cerebrovascular hemodynamics and for the protection of endothelium integrity from inflammatory, oxidative, and procoagulant stimuli. It has been demonstrated that eNOS-derived NO scavenges ROS [44] and inhibits the expression of cellular adhesion molecules [45], platelet aggregation [46], and leukocyte adhesion [47].

During ischemia, NO concentration decreases because of oxygen deficiency [41]. However, immediately after reperfusion, biosynthesis of this molecule is triggered mainly by overactivation of nNOS, as evidenced in nNOS (−/−) mice [48] or with specific inhibitors such as 7-NI [41, 49]. Glutamate-induced Ca^2+^ overload in ischemic neurons is responsible for the rise of nNOS-derived NO [50]. Concentration of NO returns to physiological levels approximately 1 h after reperfusion [48, 49, 51] and increases again due to iNOS expression between 12 h and up to 8 days later [52, 53]. iNOS sources at this stage comprise microglia [53], astrocytes [54], endothelial cells [55], and infiltrated leukocytes [56]. The amount of iNOS-NO derived is 100–1,000 times than that produced by nNOS [57].

Therefore, NO deriving from different sources (neurons, brain vessels, glia, and neutrophils) may exert an influence on the evolution of brain damage at different time-points after an ischemic insult [42]. Thus, the use of relatively selective inhibitors of NOS isoforms and genetically modified mice has contributed to clarifying the role of NO in cerebral ischemia-reperfusion injury as follows.

Total suppression of eNOS activity in knockout mice (eNOS −/−) renders them hypertensive [48] and more susceptible to ischemia-reperfusion injury, with larger infarcts compared with those of controls and a more severe reduction in cerebral blood flow (CBF) [48, 51, 58]. Conversely, overexpression of eNOS by flavonoids induces a protective effect [59]. In contrast to eNOS, infarct volume and neuronal death are consistently decreased by *nNOS* gene deficiencies or by nNOS inhibition [48, 60–64]. nNOS abolition also reduces excitotoxicity [65], nitrosative stress [63, 66], and O^2−^ production [67] and downregulates calpain and caspase-3 in ischemic lesion [61, 64]. Additionally, during reperfusion, iNOS-produced NO contributes to brain injury [42, 68]. iNOS expression is transcriptionally regulated by nuclear factor kappa B (NF-*κ*B) secondary to moderate inflammatory stimuli such as TNF-*α* [69] and IL-1*β* [70], and also by oxidative radicals [71]. Due to the large amount of NO produced by iNOS, this enzyme is related with high peroxynitrite production and significant nitrosative damage of biological molecules [72].

Consequently, nNOS mediates early neuronal injury, while iNOS contributes to late neuronal injury, whereas eNOS activity might be protective [41, 42, 73]. Whether the effects of this molecule are beneficial or harmful depends on the cellular compartment in which NO is generated, on its concentration, on the environment's redox state, and on the evolutive stage of ischemic brain injury [42, 48, 73].

In addition to their involvement in ischemia, the expression of iNOS in astrocytes and microglia and the production of large amounts of NO contribute to dopaminergic neuronal death in the neuropathology of experimental Parkinson disease [74, 75]. In the brains of patients with Alzheimer disease, nitrosylation of proteins is a hallmark of tissue damage [76] and is particularly high in hippocampus and cerebral cortex. The presence of beta amyloid is sufficient for triggering iNOS activation in astrocytes and microglia [77]. In addition, nNOS activity in reactive astrocytes surrounds beta-amyloid plaques in entorhinal cortex and is related with DNA fragmentation in CA1 and CA4 fields [78], while there is a correlation between neurofibrillary tangles and senile plaques with a reduction in eNOS capillary levels [79].

According to the dual role of NO in brain ischemia, there is a rationale for the use of NO for promoting treatments shortly after the occurrence of focal cerebral ischemia as neuroprotective strategies [42, 53, 80, 81]. The neurovascular protective mechanism of eNOS-NO suggests that intervention with NO may be most effective when delivered at an optimal amount by a suitable source at the correct time in an appropriate environment [82].

### 3.2. Nitric Oxide Donors (NOD)

Nitric oxide donors are a heterogeneous group of drugs whose common feature is the ability to release NO or an NO-related species, such as the Nitrosonium ion (NO^+^) or the Nitroxyl anion (NO^−^), *in vitro* or *in vivo*, independently on its endogenous sources [83]. The following are the NOD most frequently employed in clinical and basic research: organic nitrates (e.g., nitroglycerin, isosorbide-5-mononitrate, nicorandil, pentaerythritol tetranitrate); S-nitrosothiols (e.g., S-nitroso-N-acetylpenicillamine and S-nitroso-glutathione); sydnonimines (e.g., molsidomine, SIN-1); NONOates (JS-K, SPERMINE-NONOate, and PROLI-NONOate), and sodium nitroprusside [84].  Table 1 illustrates NOD effects on experimental cerebral ischemia.

Despite the fact that all of these are considered NOD, they have different pharmacokinetic and dynamic profiles that determine the type and extent of their biological effects. The main determinant of these effects is the manner in which NO is released, the amount of NO generated, and the time during which it is released. Moreover, some of these generate alternative products that may arise during their metabolism or decomposition. These products may even be present in quantities exceeding NO with independent or side effects [92].

For example, organic nitrates, the most common NOD utilized in coronary artery disease, require enzymatic bioactivation in order to deliver NO [93]. Their principal effect is at the vascular level, by increasing venous capacitance and coronary vasodilation [84]. S-nitrosothiols are a heterogeneous group characterized by a nitroso group attached by a single chemical bond to the sulfur atom of a thiol [83]. S-nitrosoGlutathione (GSNO) is found *in vivo* and is an important intermediary in organic nitrate metabolism. The remaining nitrosothiols are synthetic. These compounds act as NO-carriers, NO-reservoirs, and intermediates in protein nitrosylation. They also possess the ability to transfer the different NO species through chains of thiols, without releasing the NO molecule itself. This feature decreases the possibility of NO reacting with O^2−^ to form ONOO^−^, or that of reacting with other molecules to nitrosylate these [94, 95]. Sydnonimines release NO spontaneously, without enzymatic activity. Superoxide is generated concomitantly and may combine with NO to generate ONOO^−^. This process also releases significant quantities of hydroxyl radical, which increases its prooxidant potential [96]. Therefore, these compounds are considered peroxynitrite donors more than NOD and are utilized as nitrosative stress inducers in experimental models. NONOates decompose spontaneously in solution, at physiological pH and temperature, without interaction with biological molecules and in a concentration-dependent manner; thus, they usually are employed as NO-release models [90].

Sodium nitroprusside (SNP) is a compound consisting of an iron core surrounded by five Cyanide ion (CN^−^) molecules and one molecule of the Nitrosonium ion (NO^+^). SNP does not liberate NO spontaneously *in vitro*, but does require partial reduction (one-electron transfer) by a variety of reducing agents present in membrane cells. It is also possible to release NO from SNP by photolysis. In addition to NO, SNP can release, in aqueous solution, a range of oxidant and free radical species, such as iron, cyanide, superoxide, H_2_O_2_, and hydroxyl radical in direct proportion to its concentration [97–99]. Because of the nitrosative and prooxidant potential inherent in the different NOD, these have been widely used as models of neuronal damage (for a more detailed review, see [83, 84, 94]).

### 3.3. Sodium Nitroprusside-Induced Neurotoxicity

The potentially adverse effects of SNP on cells and tissues have been widely used *in vivo* and *in vitro* to study the mechanisms involved in nitrosative and OS injury. While many of the pharmacological effects elicited by SNP are attributed to the NO molecule, several *in vitro* studies [100, 101] revealed other biological SNP properties that are independent of the NO moiety, due to the huge number of by-products released during its decomposition (e.g., cyanide, iron, and ROS).

*In vitro*, SNP is usually neurotoxic. This compound is able to cause cytotoxicity in the human neuroblastoma cell line SH-SY5Y by means of OS. In addition, SNP treatment activates the (ERK1/2) pathway and inactivates the Akt pathway, leading to cell death [102]. Additionally, inhibition of ERK activation or exogenous Superoxide dismutase (SOD) treatment protects human melanoma from SNP toxicity [103]. Furthermore, in hippocampal neurons, SNP and SIN-1 are capable of decreasing Bcl-2 and increasing Bax expression along with caspase-3 activation, leading to neuronal apoptosis [104]. Concentration-dependent neuronal death was induced in cerebellar granular cells after exposure to SNP by hydroxyl radical generation, as well as by increasing the level of iron and lipid peroxidation [105]. In cultured cholinergic cells, SNP impairs oxidative metabolism of Acetyl Co-A by suppression of choline acetyltransferase and pyruvate dehydrogenase activities in mitochondria and cytoplasm. This effect triggers OS and a reduction in neuronal viability [106].

In addition to SNP, other NOD are able to elicit cytotoxicity *in vitro*. In cortical neuronal cultures, SIN-1 induced neurotoxicity by ATP depletion and protein nitration, which was counteracted by the addition of hemoglobin (a NO scavenger), SOD, and an ONOO scavenger, demonstrating that the main mediator of damage in this case is ONOO^−^ [107]. In the same cell culture type, neuronal viability was significantly reduced when compared with that of controls after DETANONOate exposure. This effect was associated with a decrease in catalase activity and expression [108].

Likewise, SNP has been used to induce neurotoxicity *in vivo*. While not mimicking a specific neuropathology, the rapid and localized neurodegeneration and demyelination caused by the SNP, when injected into the brain, provides a very practical tool for studying the role of the individual molecular players that can be involved in the immediate and consequent damage implicit in neurodegenerative processes. In animal models, SNP causes acute and localized excitotoxic cell death when infused within the brain parenchyma. This damage is also associated with a transient inflammatory response [109]. The neurodegeneration caused by SNP is accompanied by microglial activation and the induction of the proinflammatory cytokines TNF-*α* and IL-1*β*. Injection of exogenous TNF-*α* was shown to exacerbate the damage and inflammation caused by SNP through specific and transient activation of resident microglia [109]; in contrast, the abolition of the endogenous production of TNF-*α* genetically is also detrimental, because it delays microglial activation, which is later expressed in an excessive manner. However, these effects do not extend to the IL-1B. Thus, this suggests that the source, timing, and dose of TNF-*α* are preponderant in determining the fate of neurons and myelin during SNP-induced neurotoxicity [29].

Additionally, when infused into the substantia nigra, SNP induces an acute increase in lipid peroxidation, which is blocked by NO, oxyhemoglobin, and deferoxamine (an iron chelator), suggesting that OS is elicited, at least in part, by the iron moiety of SNP [110, 111]. Thus, the addition of SNP and other NOD to neuronal cultures or into brain parenchyma causes damage through the establishment of OS, nitrosative stress, and the disturbance of cellular oxidative metabolism. The death pathway activated through these mechanisms is mainly apoptotic.

However, it should be considered that the cytotoxic effects of NOD are not necessarily due to the presence of NO, because its addition to culture media alone does not cause neurotoxicity [112]; therefore, other compounds that are also part of the NOD molecules can be delivered with different effects. Such is the case of SNP, in which its toxicity lies more in its content of iron [110, 112, 113] and cyanide ions [114] rather than in its NO content or, as the case of SIN-1, whose decomposition releases superoxide anion and hydroxyl radicals along with NO, leading to the production of large amounts of ONOO^−^ [96].

**Figure 1:**
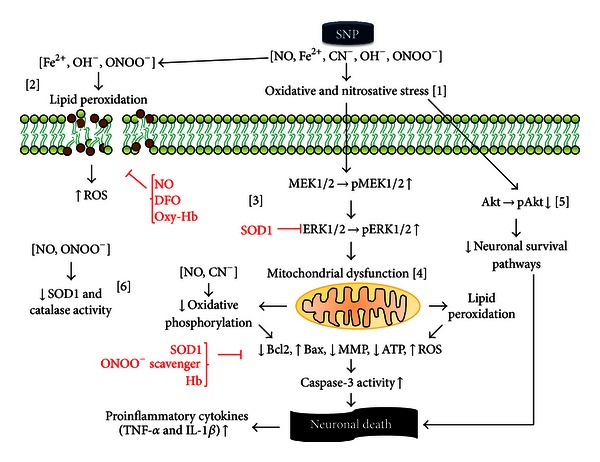
*In vitro and intracerebral effects of sodium nitroprusside and other nitric oxide donors (NOD) on neuronal survival*. SNP is capable of releasing or producing diverse byproducts, such as nitric oxide (NO), iron, cyanide anions, hydroxyl radicals, and peroxynitrite. Collectively, these are all capable of inducing oxidative and nitrosative stress [1], with the possibility of modifying the structure and function of proteins, nucleic acids, and lipids by means of oxidation and nitrosylation. Iron, via the Fenton reaction, generates OH^−^ that, together with ONOO^−^ and other reactive species, damage membranes by lipid peroxidation [2] with decreased cellular viability. This effect is blocked by the addition of NO, oxyhemoglobin, and deferoxamine, which suggests the important role of iron and NO in this reaction. The oxidative stress (OS) produced by SNP increases the activation of MEK1/2 and its substrate ERK1/2 by phosphorylation [3]. Both effects are blocked by SOD, suggesting the participation of (O^2−^) in this reaction, probably in the form of ONOO^−^. Activation of ERK1/2 is associated with a reduction of Bcl2 and an increase in (Bax), and both conditions are associated with an activation of mitochondrial apoptotic pathways. Mitochondria are a target of SNP at different levels: SNP induces lipid peroxidation of its membrane with the subsequent activation of proapoptotic pathways via caspases. In addition, NO and CN^−^ affect the functioning of the mitochondrial respiratory chain, thereby altering mitochondrial membrane potential, reducing ATP production and the generation of large amounts of reactive oxygen species [4]. The addition of ONOO^−^ scavengers and SOD1 counteracts this effect. Also, SNP decreases Akt phosphorylation [5] and reduces the expression and function of SOD1 and catalase [6]. These actions decrease antioxidant responsiveness and the activation of neuronal survival pathways. OH^−^, hydroxyl radical; ONOO^−^, peroxynitrite; Akt, protein kinase B (PKB); Bax, Bcl-2-associated X protein; Bcl2, B-cell lymphoma 2; CN^−^, cyanide anion; ERK1/2, extracellular signal-regulated kinase 1/2; IL-1*β*, interleukin 1 beta; MEK1/2, mitogen-activated protein kinase kinase 1/2; MMP, mitochondrial membrane potential; NO, nitric oxide; ROS, reactive oxygen species; SNP, sodium nitroprusside; SOD1, superoxide dismutase (Cu-Zn); TNF-*α*, tumor necrosis factor alpha.

Finally, it should be taken into account that the cytoprotective and physiological effects of NO described (e.g., vasodilatation, neurotransmission, endothelial protection) require extremely small concentrations (pico- to nanomolar), while harmful effects take place at higher concentrations, particularly under OS [93]. In culture and in intracerebral application, the NOD concentrations usually administered fall within the micro- to millimolar range. Thus, direct contact of NOD with neurons in culture or intraparenchymally coupled with the high concentration of these could be mimicking overactivation of nNOS or iNOS during the postischemic reperfusion period or in other neurodegenerative disorders [83].  Figure 1 depicts the signaling pathways involved in the neurotoxic effects of NO.

### 3.4. Nitric Oxide as an Anti-Inflammatory and Neuroprotective Agent

In contrast to the evidence presented in the previous section based on the reactivity of NO with iron and ROS, in 1994 Chiueh proposed that NO and related donor compounds may protect against the OS induced by small-molecular-weight iron complexes in the dopaminergic nigrostriatal system [115]. Since then, a growing number of reports have confirmed the potent neuroprotective and antioxidant actions of NO in the brain in experimental models of Parkinson disease [116–120]. In addition, NO has shown to inhibit lipid peroxidation of low-density lipoprotein oxidation [121, 122] in order to protect against neurotoxin-induced dopaminergic neurotoxicity [112, 118, 123, 124] and to shield cells from OS [125–127], protecting these *in vivo* through both antioxidative and -apoptotic mechanisms [118].

In the hippocampus, NO mediates cellular transduction mechanisms, regulates neuronal plasticity [128], and suppresses neuronal apoptotic cell death [129]. Thus, NO may be neuroprotective or restorative after a stroke [130–132], after traumatic brain injury [133, 134], and during Alzheimer disease [135] and depression [136].

### 3.5. NO Donors Exerted a Neuroprotective Effect against Cerebral Ischemia-Reperfusion Injury at Different Levels by Influencing Cellular Oxidative Status

SNP and SPERMINE-NONOate are able to reduce infarct size after transient focal cerebral ischemia when administered early [87]. Likewise, S-nitrosothiols (GSNO and SNAP) and SIN-1 additionally reduced infarct volume and improved neurological performance [38, 88, 89]. Hemodynamically, SNP, GSNO, and SNAP increase CBF in the penumbral region when administered at the onset of reperfusion [38, 80, 89].

In addition, pre- and postischemic administration of SNP attenuates the ischemia-induced increase of caspase-3 at 6 h of reperfusion and downregulates neuronal apoptosis by inhibiting increased phosphorylation of JNK, c-Jun, and Bcl-2 [61, 65]. This effect is achieved through nitrosylation of nNOS, which decreases its NO production. This means that SNP can regulate NO metabolism in the target cells. In focal ischemia, SNP and S-nitrosothiols decrease lipid peroxidation and nitrotyrosine formation in plasma, which is associated with less oxidative and nitrosative stress, neuroprotection, and fewer anti-inflammatory effects [38].

The effects of NOD on ischemia-reperfusion injury are also related with modulation of the inflammatory response, and these effects are probably the neuroprotective effects with the greatest impact after cerebral ischemia and reperfusion of this drug type. To shield nerve tissue from ischemia-reperfusion injury is not sufficient to protect the brain parenchyma, but also must preserve the integrity of the Blood brain barrier (BBB). Thus, the cerebral vascular endothelium is essential in the control of vascular inflammatory and oxidative responses, leukocyte migration, and the production of inflammatory mediators capable of spreading to nerve tissue [26]. Under physiological conditions, eNOS-NO is responsible for maintaining the integrity of the vascular endothelium. But under ischemic conditions, endothelial dysfunction could be offset by mimicking eNOS-derived NO neuroprotective functions by intravascular administration of a NOD [137]. Control of endothelial inflammatory and oxidative responses in turn allows restriction of their impact on resident brain cells, particularly on those with an inflammatory phenotype, such as microglia and astrocytes. Therefore, effective neuroprotection should include protection of the BBB and of the elements within it [138, 139].

*In vivo*, high expression of TNF-*α*, IL-1*β*, and iNOS in microglia and astrocytes after focal cerebral ischemia is reduced by GSNO. Likewise, GSNO induces a reduction in microglial and macrophage cells in the penumbral region, which is associated with less expression of cellular adhesion molecules such as ICAM-1 in endothelial cells [56]. Decreased expression of adhesion molecules (ICAM-1 and E-selectin) was also demonstrated with SNP and SNAP in the same model [38].

Anti-inflammatory effects are also demonstrated in other neuronal-damaged models, such as experimental autoimmune encephalomyelitis [140] and traumatic brain injury [134]. Under both conditions, NOD inhibited the expression of cell adhesion molecules and infiltration of vascular immune cells into the CNS, which subsequently led to reduction in the expression of proinflammatory cytokines at the site of injury. This suggests less damage to BBB integrity, which is an indicator of neuroprotection.

Beyond the CNS, SNP protects other organs from inflammatory damage. In cardiac surgery, SNP decreases cardiac cytokine release [141–143] and improves postischemic cardiac function [143]. In experimental models of ischemia-reperfusion injury, such as those in kidney [144] and lung [145], SNP attenuates the expression of proinflammatory cytokines and reduces leukocyte-endothelium adhesion, respectively.

### 3.6. Anti-Inflammatory Mechanisms of Nitric Oxide Donors

*In vitro* studies have elucidated some of the mechanisms involved in the anti-inflammatory effect of NOD. It is well documented that cerebral ischemia, and particularly reperfusion, leads to nuclear translocation of NF-*κ*B into the core and ischemic penumbra [146, 147], as well as into the microvessels of the affected region [148, 149]. NF-*κ*B is a key regulator of innate immunity, inflammation, and of cell survival and proliferation [150]. This inducible transcription factor is comprised of two subunits. There are five subunits that can be combined to yield homo- or heterodimers of NF-*κ*B as follows: p50, p52, c-Rel, p65 (RelA), and RelB [151]. C-Rel-containing dimer activation increases neuron resistance to ischemia [152]. Moreover, the prevalent heterodimer during cerebral ischemia and reperfusion is formed by p50- and p65-inducible subunits, and its activation contributes to the pathogenesis of postischemic injury [146, 152, 153]. NF-*κ*B is maintained in latent form in the cytoplasm of cells bound to inhibitory I*κ*B proteins. Phosphorylation of I*κ*B releases NF-*κ*B by permitting its translocation into the nucleus, its binding with NF-*κ*B motifs, and the subsequent activation of its target genes. There is, in turn, an enzymatic complex responsible for I*κ*B phosphorylation in specific serine residues, the so-called I*κ*B kinases (IKK). Activation of IKK is essential to induce NF-*κ*B activity [154].

In the ischemic brain, a wide range of stimuli may trigger activation of NF-*κ*B including, among others, the following: hypoxia [155]; IL-1, and TNF-*α* [156]; OS [157]; glutamate [158], and NOS activity, such as nNOS and iNOS [159]. Overactivation of NF-*κ*B after ischemia has been documented in neurons [147], astrocytes [53], microglia [160], and in endothelial cells [149]. Although in some hippocampal neurons NF-*κ*B have a constitutive action related with neuronal survival [150], overactivation of the p50/p65 heterodimer in neurons, glial, and endothelial cells due to ischemia, appears to contribute to acute neurodegeneration. In neurons, NF-*κ*B translocation has been associated with apoptosis [146, 147], while in glia and in vascular endothelium, NF-*κ*B activates a proinflammatory phenotype [53, 149, 160].

Therefore, blocking inflammatory phenotype activation of NF-*κ*B could disrupt the cascade of events that culminate in proinflammatory brain tissue destruction. In human endothelial cells, the addition of exogenous NO through GSNO limits TNF-*α* activation of NF-*κ*B in a time- and concentration-dependent manner [161]. This is also achieved by SNP upon stimulation with IL-1*α*, IL-1*β*, IL-4, and LPS [162]. NF-*κ*B inhibition is in fact sustained by the constitutive activity of eNOS, because its inhibition, without an inflammatory stimulus, triggers nuclear translocation of NF-*κ*B [156, 161, 162]. By inhibiting activation of the transcription factor, NO effectively blocks monocyte adhesion, as well as the expression of the proinflammatory target genes of NF-*κ*B, such as TNF-*α*, IL-6, iNOS, V-CAM, ICAM-1, E-selectin, and COX-2 [70, 156, 162–164].

In astrocytes and microglial cells, NOD also exhibits an anti-inflammatory profile through downregulation of NF-*κ*B. In primary rat astrocytes and in a BV2 microglial cell line, GSNO mitigates iNOS production by inhibiting the ability of NF-*κ*B to bind to DNA [53]. Therefore, NOD are capable not only of regulating NF-*κ*B at the vascular level, but also they possess the capability of influencing glial cell reactivity and limiting their production of iNOS, proinflammatory cytokines, and other molecules and prooxidant enzymes that are potentially harmful to neuronal cells.

Inhibition of NF-*κ*B by exogenous NO has been documented at different levels. In the brain ischemic environment, activation of NF-*κ*B occurs, at least in part, via ROS [165]. Former researches have shown that one of the most significant sources of ROS in the ischemic brain is through the metabolism of arachidonic acid by Cyclooxygenase (COX) [166, 167]. COX-2 expression is increased in brain tissue after global [168] and focal [167, 169] cerebral ischemia. ROS are produced by the peroxidase step of the COX reaction in which prostaglandin G2 is converted into prostaglandin H2 [170, 171]. Hence, reducing COX-2 activity reduces oxidative damage of the ischemic brain [167, 169]. The NO donors GSNO and SNP are able to downregulate LPS-induced COX-2 protein expression via inhibition of NF-*κ*B DNA binding activity in murine monocytes [172]. Therefore, both drugs may be candidates for neuroprotective antioxidants in cerebral ischemia. In addition, NO is a superoxide scavenger; hence, NO may inhibit NF-*κ*B by scavenging superoxide anion [162].

Furthermore, SNP is capable of interfering directly with the ability of NF-*κ*B to translocate into the nucleus. Specifically, SNP inactivates NF-*κ*B by nitration of the p65 subunit at Tyr-66 and Tyr-152. This protein modification suppresses iNOS mRNA expression and prevents the activation of NF-*κ*B target genes by TNF-*α* stimulation [164]. S-nitrosylation of the p50 subunit at Cys-62 has also been demonstrated as a major mechanism of NO regulation by inhibition of the p50 binding to its consensus DNA target sequence [173].

**Figure 2:**
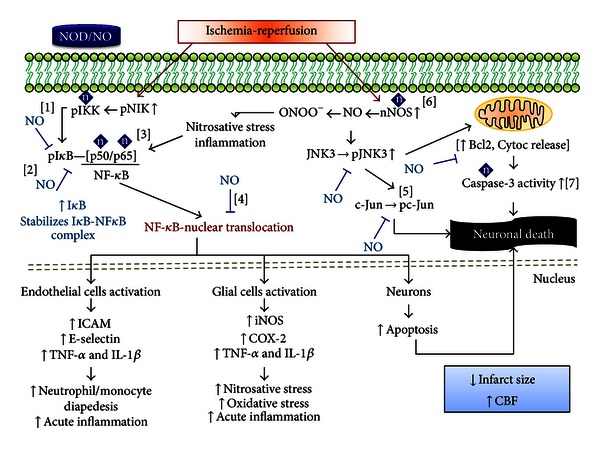
*Cerebral ischemia-reperfusion activates two major signaling pathways which exert an effect on NOD. (A) NF-*κ*B pathway*. Oxidative stress (OS) and inflammatory stimuli phosphorylate NIK. Subsequently, IKK phosphorylates NIK, which in turn phosphorylates IkB, resulting in I*κ*b degradation and NF-*κ*B translocation and activation. This action exerts different effects depending on the cell line. In endothelial cells, NF-*κ*B promotes a proinflammatory phenotype, with the expression of cellular adhesion molecules and proinflammatory cytokines that induce leukocyte migration to the ischemic territory and promote acute inflammation. In glial cells, NF-*κ*B leads to the expression of iNOS, COX-2, and proinflammatory cytokines. These effects contribute to nitrosative, oxidative, and inflammatory damage. Finally, in neurons, NF-*κ*B induces the expression of apoptosis pathways. NOD can act at different levels in this pathway: NOD-derived NO diffuses across target cell membranes, where it is able to nitrosylate kinases located upstream of NF-*κ*B, such as IKK, thereby suppressing their ability to phosphorylate [1]. This inhibition prevents I*κ*b phosphorylation and its degradation; thus the release of NF-*κ*B. NO can also increase I*κ*b gene transcription and stabilize the complex formed by I*κ*b and NF-*κ*B [2]. Furthermore, NOD-derived NO is capable of nitrosylating directly into the p50 and p65 subunits of NF-*κ*B, which blocks their ability to migrate to the nucleus [3]. All of these actions prevent the nuclear translocation of NF-*κ*B, therefore the expression of their target genes [4]. (B) *Cerebral ischemia-reperfusion increases nNOS activity, which enhances its NO production*. This NO can react with free radicals to produce ONOO^−^ and also activates the JNK3 pathway. The result is c-Jun phosphorylation and mitochondrial dysfunction, with an increase in Bcl2 phosphorylation and cytochrome C release into the cytoplasm. In addition, this activates caspase-3 and leads to neuronal apoptosis. NOD-derived NO downregulates neuronal apoptosis by inhibiting increased phosphorylation of JNK, c-Jun, and Bcl-2 [5]. This is achieved by S-nitrosylation of nNOS, which interferes with its NO production [6]. NO is also capable of nitrosylating caspase-3 directly [7]. All of these effects, along with an increase in CBF, reduce brain damage after the ischemia-reperfusion event. Bcl2, B-cell lymphoma 2; CBF, cerebral blood flow; COX-2, cyclooxygenase 2; Cytoc, cytochrome; ICAM, intercellular adhesion molecule; IkB, inhibitors of kB; IKK, I*κ*b kinase; IL-1*β*, interleukin 1 beta; iNOS, inducible nitric oxide synthase; JNK3, c-Jun N-terminal kinases 3; NF-*κ*B, nuclear factor kappa B; NIK, NF-*κ*B-inducing kinase; nNOS, neuronal nitric oxide synthase; NO, nitric oxide; NOD, nitric oxide donors; ONOO^−^, peroxynitrite; TNF-*α*, tumor necrosis factor alpha; [n] S-nitrosylation.

With respect to I*κ*B-*α*, exogenous NO increases mRNA I*κ*B levels and stabilizes the complex formed with NF-*κ*B [161, 172]. This stabilization is related with S-nitrosylation of the Cys-179 of IKKß, which decreases its ability to phosphorylate I*κ*B [174]. Additionally, NO interferes with the transient degradation of I*κ*B-*α* induced by cytokines [70]. These three actions induce negative regulation of NF-*κ*B DNA-binding activity by NOD.  Figure 2 sums up the neuroprotective actions of NOD.

## 4. Concluding Remarks

Understanding the interaction between the CNS and the immune system will provide greater insight into several different pathologies that involve CNS inflammation and the increase in the number of potential pharmacological targets.

The great variability in the observed effects elicited by NOD, from neuroprotection to toxicity, could be due to the great diversity in doses used in the experiments, which in fact are mainly distant from the existing physiological concentrations. Clarity about the NO concentrations that exists physiologically is essential for developing a quantitative understanding of NO signaling, for performing experiments with NO that emulate reality, and for knowing whether or not NO concentrations become abnormal in disease states [175]. Several independent lines of evidence suggest that NO operates physiologically at concentrations that are orders of magnitude lower than the near-micromolar order once considered correct. 

Accordingly, physiological NO concentrations range from 100 pM (or below) up to 5 nM (reviewed in [175]). Therefore, the establishment of reliable methods for directing microelectrode measurement of NO concentrations and the (most foreseeable) progression of newly developed NO biosensors for quantitative imaging of NO signaling in subcellular dimensions and in real time in tissues *in vivo* will facilitate advances in this fundamental, but yet unsettled, area.

In addition to NOD concentration, it is relevant to consider the administration pathway (intravascular, intraperitoneal, or directly into the culture media *in vitro*), as well as the cell type in which the donor exerts its action, together with the cell redox state (reduction-oxidation), because these factors are determining ones in selecting the signaling pathway that will be affected or modified by the action of these.

Therefore, therapeutic use of these molecules must be performed carefully, because they can be beneficial for one tissue or cell type and harmful for others. Given their short therapeutic window, NOD appear appropriate for use during neurosurgical procedures involving transient arterial occlusions or in very early treatment of acute ischemic stroke [87].

At present, translation from *in vitro* to *in vivo* preclinical stroke models requires further research, as clearly as that required for the case for translation from *in vivo* animal models to the clinical condition of drugs for treatment of acute ischemic stroke, which requires overcoming phase III trials in patients.

## Figures and Tables

**Table 1:** Nitric oxide donors in experimental cerebral ischemia.

Species	Model	Time of ischemia/reperfusion	Nitric oxide donor	Doses and administration	Effect	Reference
Rat	MCAO	20′/24 h	GSNO	1 mg/kg at the onset of reperfusion	Reduction in infarct size Increase in CBF Decrease in cortical expression of TNF-*α* and IL-1 in penumbral region Attenuation of iNOs expression in microglia, astrocytes and vessels of penumbral region Inhibition of monocyte/macrophage infiltration Downregulates adhesion molecules (ICAM-1, LFA-1) Reduction in TUNEL-positive cells and caspase-3 activity Blocks NF-*κ*B (p65/p50 complex) and is able to bind to DNA in astrocytes and microglial cells *in vitro *	[53]

Rat	MCAO	20′/24 h	SNP GSNO SNAP MAHMA/NONO-ate PAPA/NONOate SIN-1	2 and 3 *μ*mol/kg per 10 min IV at onset of reperfusion	Increase in CBF (except MAHMA and PAPA) Reduction in infarct size (GSNO, SNP, and SNAP) Improvement in neurological score (GSNO, SNP, and SNAP) Reduction in lipid peroxidation in plasma (all of them) Decrease in plasma levels of nitrotyrosine (GSNO, SNP and SNAP) Increase in NO plasma level (except SNAP) Reduction in mRNA expression of ICAM-1 (GSNO, SNAP, SNP) and E-selectin (except SIN-1)	[38]

Rat	MCAO	90′/24 h	ZJM-289	0.1 and 0.2 mmol/kg IV 1 h prior to ischemia	Improvement in neurological deficit (motor function) Reduction in infarct size Reduction in brain water content Decrease of neuronal degeneration Inhibition of nNOS expression Increase of NO level ipsilateral to ischemia Increase in cGMP level	[85]

Rat	MCAO	90′/1.5, 3, 4.5, 6 and 12 h	Sodium nitrite	480 nmol per 1 min at 1.5, 3, 4.5, and 6 h postischemia, IV	Reduction in infarct size (1.5, 3, 4.5 and 6 h) Improvement in motor function (4.5 h) Decrease of microhypoxic areas (12 h) Reduction in free reactive oxygen and nitrogen species (12 h)	[86]

Rat	MCAO	2 h/7 days	SNP Sperm-ine/NONO-ate	SNP: 0.11 mg/kg per 120 min, trans-ischemia, IV Spermine: 0.36 mg/kg per 120 min, trans-ischemia, IV	Reduction in infarct size Increase in cerebral perfusion	[87]

Rat	MCAO	2 h/3 days	SIN-1	0.1 and 1 mg/kg 30 min before ischemia, IV	Reduction in infarct size in normo- and hyperglycemic rats	[88]

Rat	Permanent MCAO	24 h/no reperfusion	SNP SIN-1	SNP: 3 mg/kg/h trans-ischemia, IA SIN-1: 1.5, 3, and 6 mg/kg/h trans-ischemia, IA	Both produced an increase in CBF and a reduction in infarct size SNP decreased platelet aggregation *in vitro *but not *in vivo* at the same doses	[80]

Rat	Permanent MCAO	24 h/no reperfusion	SIN-1	3 mg/kg/h per 60 min at 3, 15, 30, 60, and 120 min after ischemia, IA	Reduction in infarct size Increase in CBF	[89]

Rat	4-VO	15′/30′, 6 h, 12 h, 3 and 5 days	SNP	5 mg/kg, 3 doses: 30 min prior to ischemia, 1 h postischemia, and 2.5 h postischemia, IP	Suppression of JNK3 downstream pathway (30′, 3 h) Increase in Akt and Bad phosphorylation (12h) Inhibition of Cytochrome c release from mitochondria (6 h) Reduction in TUNEL-positive cells and caspase-3 activity (3 h) Augmentation of neuronal survival in CA1 pyramidal layer (3–5 d)	[61]

Rat	4-VO	15′/6 h, 3 and 5 days	SNP	5 mg/kg, 3 doses: 30′ prior to ischemia, 1 h postischemia and 2.5 h postischemia, IP	Decreased hippocampal activation of nNOS by nitrosylation and phosphorylation (6 h) Suppression of JNK3 downstream pathway (6 h) Inhibition of release of Cytochrome C into cytoplasm (6 h) Attenuation of caspase-3 activity (6 h) Reduction in neuronal degeneration (5 d) and TUNEL-positive cells (3 d) in CA1 pyramidal layer	[65]

Rabbit and rat	MCAO	60′/2, 4 h respectively	ProliNO/ NONO-ate	Rabbit: 10^−6^ mol/L Rat: 10^−5^ mol/L At the onset of reperfusion per 60 min, IA	Reduction in free reactive oxygen species Reduction in infarct size	[90]

Goat	MCAO	20′/7 days	SNP DEA/NONOate DETA/NONOate	SNP: 10^−9^–3 × 10^−4^ mol/L, IV DEA: 10^−9^–3 × 10^−4^ mol/L, IV DETA: 10^−7^–3 × 10^−4^ mol/L, IV	MCA relaxation	[91]

Lines of evidence are ordered first by animal model and then by surgical procedure and severity of the ischemia. In cases in which the effects were different at different reperfusion times, this is indicated after each effect by the corresponding time as a superscript between parentheses. MAP: Mean arterial pressure; CA1: Cornu Ammonis; CBF: Cerebral blood flow; GSNO: S-nitrosoGlutathione; IA: IntraArterial; ICAM-1: Intercellular adhesion molecule-1; IL1: Interleukin 1; iNOS: inducible Nitric oxide synthase; IP: Intraperitoneal; IV: Intravenous; JNK3: c-Jun N-terminal kinase-3; LFA: Lymphocyte function-associated antigen-1; MAHMA: Methylamine hexamethylene methylamine NONOate; MCA: Middle cerebral artery; MCAO: Middle cerebral artery occlusion; nNOS: neuronal Nitric oxide synthase; NO: Nitric oxide; PAPA: Propylamine propylamine NONOate; SAP: Systolic arterial pressure; SIN-1: 3-morpholinoSydnonimine; SNAP: S-nitroso-N-acetyl-penicillamine; SNP: Sodium nitroprusside; TNF: Tumor necrosis factor; TUNEL: Terminal dUTP nick end labeling; 4-VO: four Vessel occlusion model.
